# Decomposing PPI networks for complex discovery

**DOI:** 10.1186/1477-5956-9-S1-S15

**Published:** 2011-10-14

**Authors:** Guimei Liu, Chern Han Yong, Hon Nian Chua, Limsoon Wong

**Affiliations:** 1 School of Computing, National University of Singapore, Singapore; 2 NUS Graduate School for Integrative Sciences and Engineering, National University of Singapore, Singapore; 3 Data Mining Department, Institute for Infocomm Research, Singapore

## Abstract

**Background:**

Protein complexes are important for understanding principles of cellular organization and functions. With the availability of large amounts of high-throughput protein-protein interactions (PPI), many algorithms have been proposed to discover protein complexes from PPI networks. However, existing algorithms generally do not take into consideration the fact that not all the interactions in a PPI network take place at the same time. As a result, predicted complexes often contain many spuriously included proteins, precluding them from matching true complexes.

**Results:**

We propose two methods to tackle this problem: (1) The localization GO term decomposition method: We utilize cellular component Gene Ontology (GO) terms to decompose PPI networks into several smaller networks such that the proteins in each decomposed network are annotated with the same cellular component GO term. (2) The hub removal method: This method is based on the observation that hub proteins are more likely to fuse clusters that correspond to different complexes. To avoid this, we remove hub proteins from PPI networks, and then apply a complex discovery algorithm on the remaining PPI network. The removed hub proteins are added back to the generated clusters afterwards. We tested the two methods on the yeast PPI network downloaded from BioGRID. Our results show that these methods can improve the performance of several complex discovery algorithms significantly. Further improvement in performance is achieved when we apply them in tandem.

**Conclusions:**

The performance of complex discovery algorithms is hindered by the fact that not all the interactions in a PPI network take place at the same time. We tackle this problem by using localization GO terms or hubs to decompose a PPI network before complex discovery, which achieves considerable improvement.

## Introduction

High-throughput experimental techniques have produced large amounts of protein interactions, which makes it possible to discover protein complexes from protein-protein interaction (PPI) networks. A PPI network can be modeled as an undirected graph, where vertices represent proteins and edges represent interactions between proteins. Protein complexes are groups of proteins that interact with one another, so they are usually dense subgraphs in PPI networks. Many algorithms have been developed to discover complexes from PPI networks [1-8].

As a model organism, *Saccharomyces cerevisiae* (baker’s yeast) has been extensively studied, and its PPI network is now relatively complete. However, the performance of existing complex discovery algorithms on the yeast PPI network is not very satisfactory. One reason behind this is that each protein do not necessarily participate in all its known interactions simultaneously. With very few exceptions [9], existing complex discovery algorithms generally do not take this into consideration. As a result, the clusters generated often contain extra proteins that preclude them from matching true complexes. An ideal solution would be to decompose the PPI network into several smaller networks such that interactions within each smaller network are contextually coherent. In reality, it is very difficult to know which subset of interactions take place together. Here we choose to use cellular component GO terms to decompose PPI networks because a protein complex can be formed only if its proteins are localized within the same compartment of the cell. We use only localization GO terms that are relatively general for decomposition. The existence of hub proteins is another factor that makes it difficult for complex discovery algorithms to decide the boundary of clusters. Hub proteins are proteins that have a lot of neighbors in the PPI network, and these neighbors often belong to multiple complexes [10]. This may fuse clusters that correspond to different complexes. To avoid this, we remove hub proteins from PPI networks prior to clustering. After the clusters are generated from the remaining PPI network, we then add the removed hub proteins back to the clusters.

We tested the above methods on the yeast PPI network downloaded from BioGRID [11]. The results show that these methods can improve the performance of existing complex discovery algorithms significantly. A preliminary version of this paper was presented as a short paper [12] in BIBM2010. In this version, we have included more experimental results and further discussed why some complexes are so hard to detect. In the rest of the paper, we first describe the two methods for decomposing PPI networks, and then show experiment results.

## Methods

In this section, we first describe the two methods for decomposing PPI networks for complex discovery, and then briefly introduce the complex discovery algorithms used in our experiments.

### The localization GO term decomposition method

A protein complex can only be formed if its proteins are localized within the same compartment of the cell. Hence we use cellular component GO terms to decompose a given PPI network into several smaller PPI networks such that all proteins in each smaller network are annotated with the same localization GO term. We use only localization GO terms that are relatively general for decomposition. There are several reasons for this. First, it is relatively easy to obtain the rough localization of proteins, compared with obtaining the precise and specific localization of proteins. Secondly, very specific GO terms are annotated to very few proteins. Using them to decompose PPI networks produces many small fragments, and lots of information may be lost due to the decomposition. Finally, some very specific cellular component GO terms correspond to complexes, and they are just as hard to decide as complexes.

We use a threshold *N_GO_* to select GO terms for decomposition, where *N_GO_* should be large. The selected GO terms are annotated to at least *N_GO_* proteins, and none of their descendant terms is annotated to at least *N_GO_* proteins. If a GO term is selected, then none of its ancestor terms or descendant terms will be selected.

Given a selected GO term, we first remove all the proteins that are not annotated to the term from the given PPI network, and then apply a complex discovery algorithm on the resultant network. This process is repeated for every selected GO term. The final set of clusters is the union of the clusters discovered from every filtered network. Duplicated clusters are removed.

### The hub removal method

Hub proteins are those proteins that have many neighbors in the PPI network. We use a threshold *N_hub_* to define hub proteins. We call a protein a *hub protein* if it has at least *N_hub_* neighbors. A hub protein often connects proteins that belong to different complexes, which makes it hard to decide the boundary of the complexes and the membership of the hub proteins.

To alleviate the impact of the hub proteins, we first remove hub proteins from a given PPI network, and then use an existing complex discovery algorithm to find clusters from the remaining network. After the clusters are generated, hub proteins are added back to the clusters. We add a hub protein *u* back to a cluster *C* based on the connectivity between *u* and *C*, which is defined as follows:(1)

where *w*(*u*,*v*) is the weight of edge (*u*,*v*), and it is calculated from the original PPI network using iterative AdjustCD [8] before removing hubs. If there is no edge between *u* and *v*, then *w*(*u*, *v*)=0. A hub protein *u* is added to a cluster *C* only if *Connectivity*(*u*, *C*) ≥ *hub_add_thres*, where *hub_add_thres* is a number between 0 and 1.

### Combining the two methods

We combine the two methods by first removing hub proteins from the given PPI network, and then decomposing the resultant PPI network using selected GO terms. The whole process is described below:

1. Let  be the set of clusters generated. Initially  is empty.

2. Remove hub proteins that have at least *N_hub_* neighbors from the given PPI network *G*. Let *G′* be the resultant network.

3. Let *g*_1_,⋯,*g_m_* be the localization GO terms that are selected using threshold *N_GO_*. For each *g_i_*, do the following:

• Remove proteins that are not annotated with *g_i_* from *G′*. Let  be the resultant network.

• Apply a complex discovery algorithm on  to find clusters. Let  be the set of clusters generated.

• ;

4. Remove duplicated clusters from .

5. Add hub proteins back to clusters in .

### Complex discovery algorithms

We used the following complex discovery algorithms in our study. MCL and RNSC generate a partition of the PPI network, and they do not allow overlap among clusters. The other two algorithms, IPCA and CMC, allow overlap among clusters.

Markov Cluster Algorithm (**MCL**) [1] is motivated by a heuristic formulated in terms of stochastic flow. It iteratively enhances the contrast between regions of strong and weak flow in the graph. The process converges towards a partition of the graph, with a set of high-flow regions (the clusters) separated by boundaries with no flow. The performance of MCL is mainly affected by the“-I inflation” option, which controls the granularity of the output clustering.

Restricted Neighborhood Search Clustering (**RNSC**) [13] is a cost-based local search algorithm that explores the solution space to minimize a cost function, calculated according to the number of intra-cluster and inter-cluster edges. RNSC searches for a low-cost clustering by first composing an initial random clustering, and then iteratively moving a node from one cluster to another in a randomized fashion to reduce the clustering’s cost. It also makes diversification moves to avoid local minima. RNSC performs several runs, and reports the clustering from the best run. The number of runs is controlled by the “-e” option.

**IPCA**[7] follows the general approach of cluster expanding based on seeded vertices. It first assigns weights to edges and vertices, and then picks the vertex with the highest weight as the seed of a new cluster. Other vertices are then added to the cluster based on their connectivity. For each of the subsequent cluster, the vertex with the highest weight among those vertices that do not appear in previous clusters is chosen as the seed, and the cluster is expanded using all the vertices except those seed vertices in the previous clusters. Whether a vertex can be added to a cluster is determined by the diameter of the resultant cluster (the “-P” option) and the connectivity between the vertex and the cluster (the “-T” option).

Clustering by Maximal Cliques (**CMC**) [8] first generates all the maximal cliques from a given PPI network, and then removes or merges highly overlapping cliques based on their inter-connectivity as follows. Each maximal clique is assigned a score based on their weighted density and size. If the overlap between two maximal cliques exceeds a threshold *overlap_thres*, then CMC checks whether the inter-connectivity between the two cliques exceeds a threshold *merge_thres*. If it does, then the two cliques are merged together; otherwise, the clique with lower score is removed.

## Results and discussion

In this section, we first describe the datasets and the evaluation method used in our experiments, and then study the impact of the two decomposition methods on the performance of the four complex discovery algorithms.

### Experiment settings

#### PPI data

We used the yeast PPI dataset downloaded from BioGRID [11] (version 3.0.64) in our experiments. We kept only physical interactions that are generated by the following experiment types: *Affinity Capture-Luminescence*, *Affinity Capture-MS*, *Affinity Capture-RNA*, *Affinity Capture-Western*, *Biochemical Activity*, *Co-crystal Structure*, *Co-fractionation*, *Co-localization*, *Co-purification*, *Far Western*, *FRET*, *PCA*, *Protein-peptide*, *Protein-RNA*, *Reconstituted Complex*, *Two-hybrid*. Self-interactions are removed. The dataset contains 5765 proteins and 52096 binary interactions.

#### Evaluation methods

We match the generated clusters with reference complex sets, and calculate recall (sensitivity) and precision. Let *S* be a cluster, *C* be a reference complex, *V_S_* and *V_C_* be the set of proteins contained in *S* and *C* respectively. We define the matching score between *S* and *C* as the Jaccard index between *V_S_* and *V_C_*.(2)

Given a threshold *match_thres*, if *match_score*(*S*, *C*) ≥ *match_thres*, then we say *S* and *C* match each other. Given a set of reference complexes  and a set of predicted complexes , recall and precision are defined as follows:(3)(4)

There is often an inverse relationship between precision and recall. We combine the two measures into a single measure called F1-measure to assess the overall performance. F1-measure is defined as follows:(5)

#### Reference complexes

Three reference sets of protein complexes are used in our experiments. Two set of complexes are hand-curated complexes from MIPS [14] and the CYC2008 catalogue [15]. The third set is generated by Aloy et al. [16]. We combine these three sets of complexes together, and keep only those complexes with size no less than 4. Duplicated complexes are removed. Table 1 shows the number of complexes, number of proteins, the maximal, average and median size, and the average and median density of the complexes in the three reference complex sets and the combined set. In all the experiments below, we used the combined reference complex set, and considered complexes and clusters with size no less than 4.

**Table 1:** Statistics of reference complexes

Datasets	#cmplx	#proteins	max size	avg size	median size	avg density	median density
Aloy	63	544	34	9.22	7	0.865	0.944
CYC08	148	1115	81	8.84	6	0.831	0.944
MIPS	156	1171	95	14.86	9	0.565	0.564
Combined	305	1543	95	11.85	7	0.697	0.800

Only complexes of size ≥4 are considered.

#### Parameter settings of the four complex discovery algorithms

Unless stated explicitly, the parameters of the four complex discovery algorithms are set as in Table 2. Parameters not shown are set to their default values.

**Table 2:** Parameter settings of complex discovery algorithms

algorithms	parameter settings
MCL	-I 1.8
RNSC	-e10 -D50 -d10 -t20 -T3
IPCA	-T0.4
CMC	*overlap_thres*=0.5, *merge_thres*=0.4

### Results of the GO term decomposition method

The first experiment studies the impact of the GO term decomposition method on the performance of the four algorithms. We use annotations in Gene Ontology [17] (dated 4 June, 2010) to select GO terms for decomposition. Table 3 shows the number of GO terms selected under different *N_GO_* values. If a protein is annotated to none of the selected GO terms, then the protein is discarded because it does not occur in any of the small PPI networks after decomposition. The number of such proteins is shown in the third column. If the two proteins of an interaction do not share a common selected GO term, then the two proteins do not occur in any common PPI network after decomposition and the interaction between them is lost too. The number of such interactions is shown in the last column of Table 3. The number of proteins and interactions that are discarded is considerably large when *N_GO_* is small.

**Table 3:** Number of GO terms selected under different *N_G__O_* values

*N_GO_*	#GO terms selected	#proteins discarded	#PPIs discarded
1000	6	2067	27151
500	10	2193	27477
300	10	2481	33425
100	29	3023	39992
30	57	3461	43638

**Figure 1:**
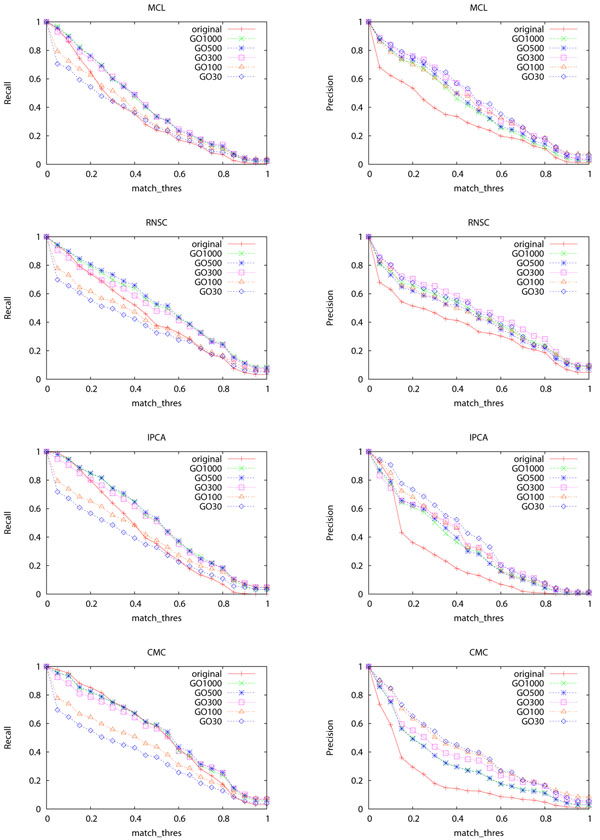
**Recall and precision of the four algorithms when different sets of GO terms are used for decomposition** The X-axis is *match_thres*. “GOn” means that the GO terms are selected using a threshold of *n*. For example, “GO1000” means that the GO terms are selected using a threshold of 1000. “original” means that complex discovery is performed on the original network without decomposition.

Figures 1 shows the recall and precision of the four complex discovery algorithms when different *N_GO_* thresholds are used for selecting localization GO terms. The precision of all the four algorithms is improved considerably under all the different *N_GO_* values. The recall is improved as well when *N_GO_* ≥ 300. When *N_GO_*=30 or 100, recall of the four algorithms decreases. This is mainly because too much information is lost in these two cases as shown in Table 3. Hence we should use GO terms that are relatively general to decompose PPI networks to avoid breaking the whole network into tiny fragments. Overall, the performance of all the four algorithms improves. We have also tested other parameter settings of the four complex discovery algorithms besides that shown in Table 2. The improvements achieved are all very similar.

**Figure 2:**
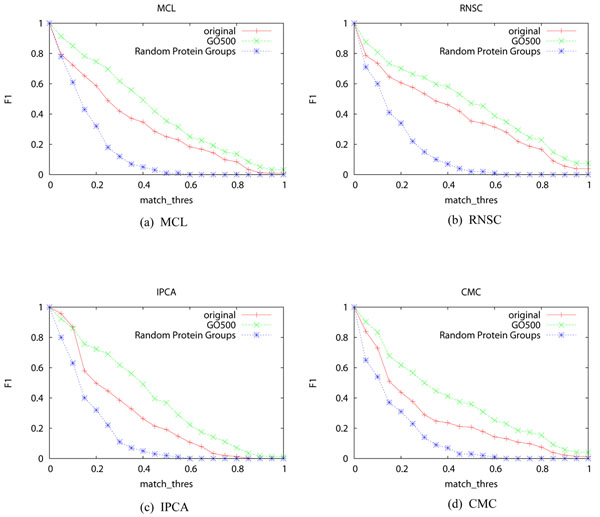
F1-measure of the four algorithms when random protein groups are used for decomposition

We also compared the above improvement with that of using random protein groups for decomposition. Random protein groups are generated by replacing proteins of the selected GO terms with randomly picked proteins. We generated 100 sets of random protein groups and use their mean F1-measure as the result. Figures 2 shows that using random protein groups to decompose the PPI network decrease the performance of the four algorithms greatly, where the random protein groups were generated from GO terms selected at a threshold of 500.

### Results of the hub removal method

The second experiment studies the impact of the hub removal method on the performance of the four algorithms. Table 4 shows the number of hub proteins and interactions removed under different *N_hub_* values. The numbers indicate that a small number of hub proteins account for a large number of interactions. For example, the percentage of proteins with at least 100 neighbors is less than 2%, while they account for about 37% of the interactions.

**Table 4:** #hub proteins and #PPIs removed under different *N_hub_*

*N_hub_*	#hub proteins removed	#PPIs removed
100	97	19292
75	207	26331
50	446	35632
40	651	40534
30	996	45568
20	1550	49775

We use parameter *hub_add_thres* to determine when a hub can be added to a cluster. In our experiments, we found that the proper range for *hub_add_thres* is [0.2, 0.9]. In the rest of the experiments, we set *hub_add_thres* to 0.3.

**Figure 3:**
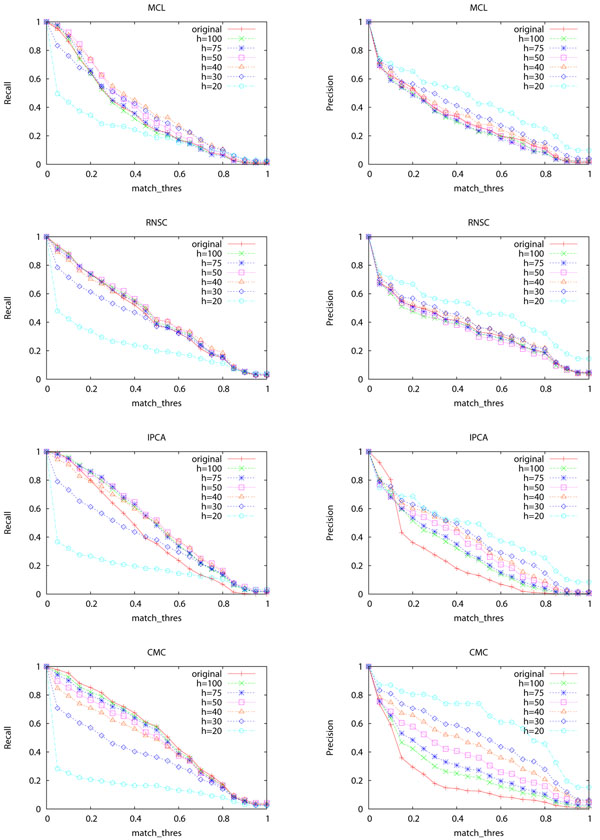
**Recall and precision of the four algorithms when different *N_hub_* values are used for removing hubs** The X-axis is *match_thres*. “hubn” means that a value of *n* is used to define hub proteins. For example, “hub100” means proteins with at least 100 neighbors are regarded as hubs. “original” means that complex discovery is performed on the original network without hub removal.

Figures 3 shows the recall and precision of the four complex discovery algorithms when different *N_hub_* thresholds are used for removing hub proteins. The recall of the four algorithms decreases greatly when *N_hub_* ≤ 30, which indicates that too many proteins and interactions are removed as shown in Table 4. The hub removal strategy is not helpful for RNSC and MCL, but is very helpful for IPCA and CMC. The main improvement of IPCA and CMC is on precision.

It has been proposed that two types of hubs exist: party hubs that interact with all their neighbours simultaneously, and date hubs that interact with different neighbours at different times [10]. We postulate that when *N_hub_* ≥ 30, most of the hubs removed correspond to date hubs, as it is physically unlikely for a protein to bind to so many other proteins at the same time due to its limited surface area. However, when removing hubs with fewer neighbours, it might be helpful to identify and remove only date hubs, while ignoring party hubs. To test this hypothesis, we removed only hubs that are part of at least 3, 5, or 7 reference complexes, for *N_hub_* =5-9, 10-14, or 15-19. This experiment assumes that we have a classifier which is able to accurately distinguish between date hubs (hubs that belong to many reference complexes) and party hubs (hubs that belong to fewer complexes). However, none of these settings show any significant improvement over not removing these hubs with fewer neighbours, possibly because too few hubs were removed to have a significant impact on performance.

### Results of combining the two methods

**Figure 4:**
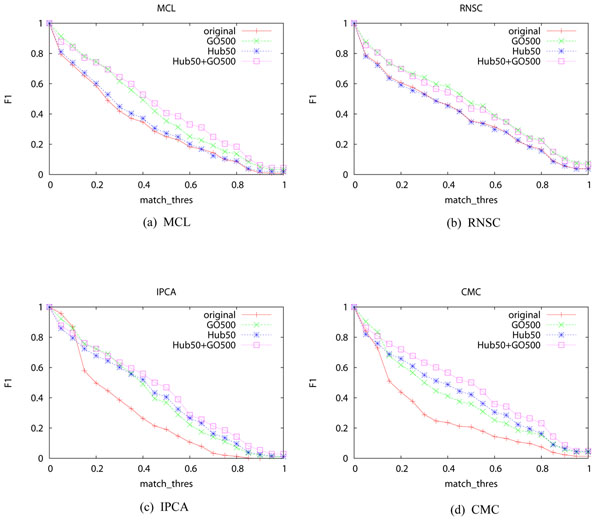
**F1-measure of the four algorithms when the two methods are performed in tandem** The X-axis is *match_thres*. “original” means the original network with neither hub removal nor GO term decomposition. “GO500” means that the network is decomposed using GO terms selected at a threshold of 500. “Hub50” means that hub proteins with at least 50 neighbors are removed. “Hub50+GO500” means that first hub proteins with at least 50 neighbors are removed, and the network is then decomposed using GO terms selected at a threshold of 500.

The last experiment is to examine the combined impact of the two decomposition methods. We set *N_GO_* to 500 and *N_hub_* to 50. Figures 4 shows the results. RNSC and MCL do not benefit much from the hub removal method, so for these two algorithms, combining the two decomposition methods yields little improvement compared with using GO decomposition alone. The performance of IPCA and CMC improve when both methods are used.

Table 5 shows the F1 of the four algorithms when *match_thres*=0.50. RNSC shows the best performance on the original PPI network, while CMC performs the best when the two decomposition methods are used.

**Table 5:** F1-measure of the four algorithms when *match_thres*=0.5

	original	Hub50	GO500	Hub50+GO500
MCL	0.250	0.272	0.354	0.406
RNSC	0.353	0.347	0.471	0.436
IPCA	0.191	0.405	0.368	0.469
CMC	0.207	0.421	0.359	0.501

## Discussion

**Figure 5:**
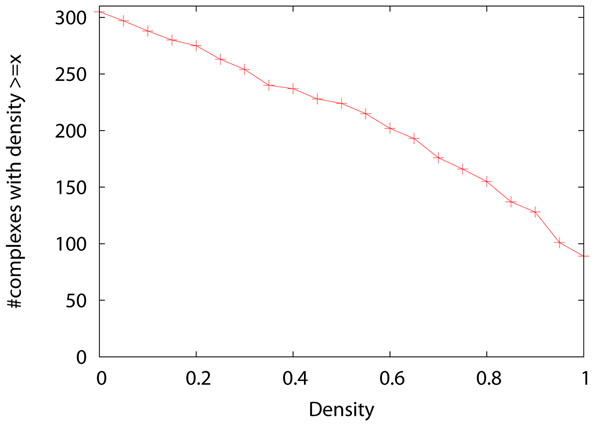
**Density of reference complexes** The X-axis is density. The Y-axis is the number of reference complexes with density ≥ *x*.

**Figure 6:**
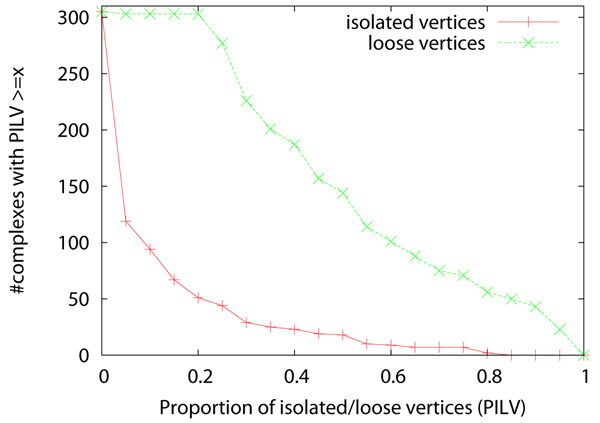
**Distribution of isolated/loose vertices in complexes** The X-axis is the proportion of isolated/loose vertices in a complex. The Y-axis is the number of complexes with proportion of isolated/loose vertices >= *x*.

**Figure 7:**
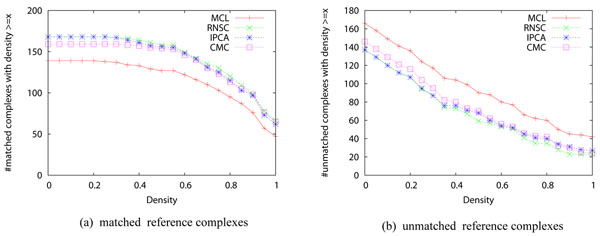
**Density of matched/unmatched reference complexes** The X-axis is density. The Y-axis is the number of detected/undetected reference complexes with density ≥ *x*. The matching threshold is set to 0.50.

Even though the performance of the four algorithms improves significantly after applying the two pre-processing methods, many reference complexes remains undetected by all four algorithms. On the original network, 116 reference complexes cannot be detected by any of the four complex discovery algorithms when *match_thres*=0.5. This number reduces slightly to 113 after applying the two pre-processing methods. To find out why these complexes cannot be detected, we study the density of the reference complexes and the connectivity of the vertices in the complexes. We define a vertex in a complex as an *isolated vertex* if it connects to none of the other vertices in the same complex. We define a vertex in a complex as a *loose vertex* if it connects to less than half of the other vertices in the same complex. Complexes with low density or containing many isolated/loose vertices are very difficult to detect. Figure 5 shows the density of the complexes. Figure 6 shows the proportion of isolated vertices and loose vertices in the complexes. Among the 305 complexes, 81 complexes have a density less than 0.5, and 42 complexes have a density less than 0.25. There are 144 complexes with more than half of their proteins being loose proteins, and these complexes are not easy to detect. There are 18 complexes with more than half of their proteins being isolated vertices, and these complexes are almost impossible to discover. Figure 7 shows the density of the complexes that are detected or are not detected separately. Most of the complexes that are detected have high density. For all the four algorithms, 90% of the detected complexes have a density no less than 0.5. On the contrary, many complexes that are not detected have a density less than 0.5, and they also have many loose vertices. There are some complexes that have very high density but cannot be discovered by the four algorithms. We found that for such complexes, usually there exists some cluster such that the complex is a subset of the cluster but the size of the cluster is too large to qualify as a match. If we lower the matching threshold to 0.33, then most of these high density complexes can be matched by some clusters.

## Conclusions

In this paper, we proposed two methods to decompose PPI networks for complex discovery. We used four complex discovery algorithms to experimentally study the effectiveness of the two methods. The results show that the two decomposition methods help improve the performance of the four algorithms significantly. The two partitioning clustering algorithms, MCL and RNSC, benefit more from the GO decomposition method, while the two algorithms that allow overlap among clusters, CMC and IPCA, benefit from both.

For the GO term decomposition method, we recommend using localization GO terms that are relative general because their annotations are easier to obtain and they also preserve more information than GO terms that are very specific.

There are two main reasons why some complexes cannot be detected. First, there might be too few interactions existing between proteins in the complex. Secondly, the complex itself might be densely connected, but so is the region surrounding it, which makes it difficult to correctly delineate the boundary around it. Both cases are difficult to handle. We may need to use other information besides PPI data to detect such complexes.

## Competing interests

The authors declare that they have no competing interests.

## Authors' contributions

GL conducted the experiments and wrote this manuscript. CHY and HNC gave suggestions on the proposed methods as well as the performance study. They also helped improve the manuscript. LW guided the study and improved the writing of the manuscript. All authors read and approved the final manuscript.
